# SVPath: A Deep Learning Tool for Analysis of Stria Vascularis from Histology Slides

**DOI:** 10.1007/s10162-024-00948-z

**Published:** 2024-05-17

**Authors:** Aseem Jain, Dianela Perdomo, Nimesh Nagururu, Jintong Alice Li, Bryan K. Ward, Amanda M. Lauer, Francis X. Creighton

**Affiliations:** 1 College of Medicine, University of Cincinnati, 231 Albert Sabin Way, Cincinnati, OH 45267 USA; 2 Department of Otolaryngology-Head and Neck Surgery, Johns Hopkins University School of Medicine, Baltimore, MD USA

**Keywords:** Stria vascularis, Deep learning, Artificial intelligence, Temporal bone histology

## Abstract

**Introduction:**

The stria vascularis (SV) may have a significant role in various otologic pathologies. Currently, researchers manually segment and analyze the stria vascularis to measure structural atrophy. Our group developed a tool, SVPath, that uses deep learning to extract and analyze the stria vascularis and its associated capillary bed from whole temporal bone histopathology slides (TBS).

**Methods:**

This study used an internal dataset of 203 digitized hematoxylin and eosin-stained sections from a normal macaque ear and a separate external validation set of 10 sections from another normal macaque ear. SVPath employed deep learning methods YOLOv8 and nnUnet to detect and segment the SV features from TBS, respectively. The results from this process were analyzed with the SV Analysis Tool (SVAT) to measure SV capillaries and features related to SV morphology, including width, area, and cell count. Once the model was developed, both YOLOv8 and nnUnet were validated on external and internal datasets.

**Results:**

YOLOv8 implementation achieved over 90% accuracy for cochlea and SV detection. nnUnet SV segmentation achieved a DICE score of 0.84–0.95; the capillary bed DICE score was 0.75–0.88. SVAT was applied to compare both the ears used in the study. There was no statistical difference in SV width, SV area, and average area of capillary between the two ears. There was a statistical difference between the two ears for the cell count per SV.

**Conclusion:**

The proposed method accurately and efficiently analyzes the SV from temporal histopathology bone slides, creating a platform for researchers to understand the function of the SV further.

## Introduction

The stria vascularis (SV) is a vascular structure adjacent to the lateral wall of the cochlea that plays a critical role in audition through the secretion of endolymph, maintenance of endocochlear potential, and formation of the blood-labyrinth barrier within the inner ear [1]. The structure primarily comprises three major cell types: marginal, intermediate, and basal. It also includes a capillary bed and other less common cells, such as spindle and endothelial cells [2–5].

Since the 1900s, various otologic pathologies have been linked to dysfunction of the SV [2]. Carraro et al. and other groups have found marked atrophy of the SV and its capillary bed in patients with presbycusis, and additional studies have shown how morphologic changes of the SV occur with noise-induced and autoimmune hearing loss [3–7]. Notably, in the mid-1900s, autonomic dysregulation linked to vasospasms in the stria and surrounding vasculature was linked to Meniere’s disease, and the structure is still thought to play a role in cytomegalovirus-induced neonatal hearing loss and sudden sensorineural hearing loss [3–5, 8].

Researchers have assessed histologic slides of the inner ear from deceased humans and other mammals to understand how pathologic processes affect different microstructures for a century [9]. Most histological studies of the inner ear, including those of the SV and its capillary bed, have been done manually, annotating and segmenting these structures from slides [5, 7, 9, 10]. Manual annotation is time consuming and subject to inter-rater variability [10]. These practical constraints may hinder the discovery of SV-related changes associated with human disease.

In recent years, advancements in digital microscopy have facilitated the curation of whole-slide pathology imaging (WSI) databases. Researchers have begun applying deep learning techniques to these databases to automate the analysis of structures from histology slides [11–13]. Analysis and segmentation of the SV from temporal bone WSI pose many practical challenges. WSI files are large, often in the order of 1–2 gigabytes per image. Deep learning methods applied to these large files are computationally expensive and slow to train and implement [11]. Their performance diminishes with inconsistent staining of the slides and insufficient training labels [12]. Further, deep learning methods can have difficulty accurately segmenting small structures like the SV within large slides. For example, in terms of pixels, the SV is less than a millionth of the entire WSI.

Our aim in this paper is to propose a deep learning tool, SVPath, for automatic analysis of the SV from WSI. To resolve some of the challenges outlined above, we split this task into three subtasks: detecting the SV within the WSI, segmenting the SV and its capillary bed, and extracting relevant metrics from the segmentation. We then validate these subtasks by applying this technique to slides from two ear WSI from different macaques.

## Methods

### Dataset

The dataset used to develop this tool (SVPath) comprises 203 temporal bone histology sections from a normal macaque ear, M1. A separate normal macaque ear, M2, was also used to validate the SVPath tool further. Ten-micron thick slices were sequentially sectioned from the temporal bones parallel to the plane of the superior semicircular canal for both ears resulting in around 500 slices for each ear. The slices were then scanned using an Olympus microscope at × 20 magnification. Each scanned image was downsampled by 4 × and saved in the tiff image format using QuPath [14]. The average size of a single image was ~ 500 megabytes.

### Deep Learning Method to Segment Stria Vascularis and Capillary Bed

**Fig. 1:**
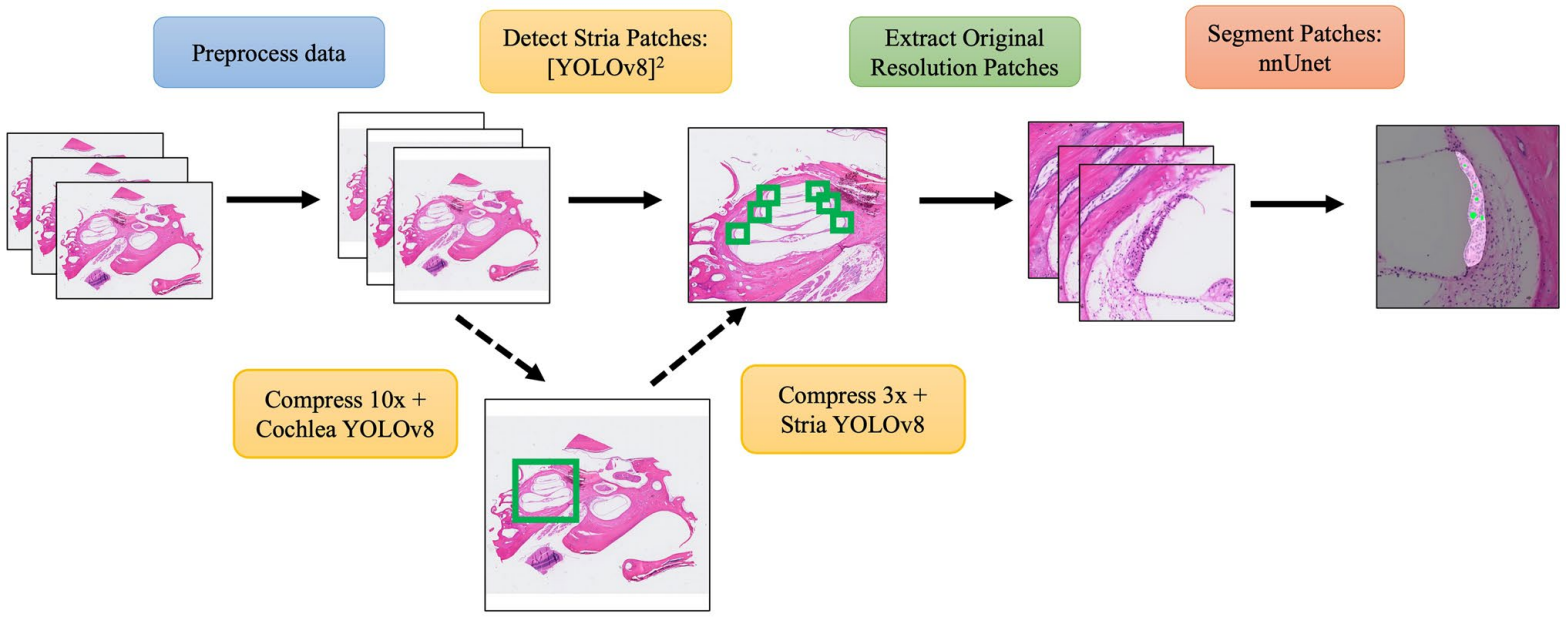
Overview of the method to extract stria vascularis and its associated capillaries from whole slide imaging

An overview of the proposed method used to segment and analyze the SV automatically is shown in Fig. 1. Before using any deep learning method, in the preprocessing block, blank pixels were added to the borders of each tiff image (~ 13,000 × 16,000 pixels) to create square image represented by 18,000 × 18,000 pixels (px) for image standardization. The proposed algorithm uses two neural network architectures: YOLOv8 and nnUnet. YOLOv8 is a recent modification to the original YOLO framework: an efficient object detection neural network used to extract patches of images where features are present [15]. nnUnet is an improvement on a traditional U-net: a fully convolutional neural network that can perform semantic or pixel-level segmentation of structures [16]. Each of these models is implemented with PyTorch and other Python libraries [16]. The training process for these models is described below.

After the data is preprocessed into uncompressed square images, it is incorporated into the trained [YOLOv8]^2^, which consists of two individual YOLOv8 neural networks to extract features at the level of the WSI and then at the level of the cochlea. The uncompressed square WSI is first compressed into a 2048 × 2048 px image that is then processed in the first YOLOv8 to determine the location of the cochlea within the WSI. The location of the cochlea in this compressed image is mapped to the uncompressed square WSI and used to extract an uncompressed square region (9000 × 9000 px) centered around the cochlea. Like the initial step, this region is compressed and input into the second YOLOv8 neural network to determine the location of SV within the uncompressed region around the cochlea. From this location, 512 × 512 px patches centered around each SV are extracted and stored in a separate folder. This method overcomes the limitation of extracting small features from WSI while preserving the original uncompressed image. The 512 × 512 patches are then analyzed separately by nnUnet to extract binary masks of the SV and its associated capillary bed. These masks are stored and used in the SV Analysis Tool (SVAT) described below.

### Stria Vascularis Analysis

**Fig. 2:**
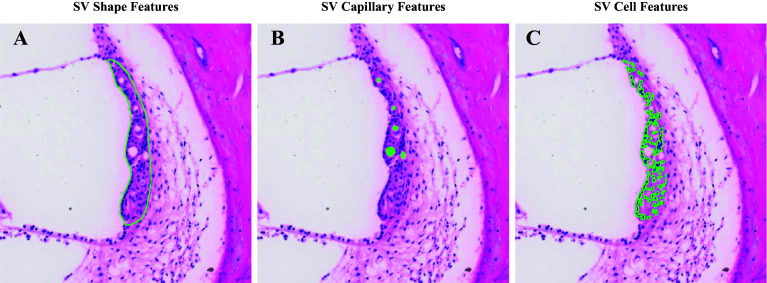
Overview of features extracted from stria vascularis (SV). **A** Outline of SV; overall area and width were computed from this region. **B** Capillary regions in SV are highlighted; the average area of capillary in SV and total capillary area per SV were extracted. **C** Outline of nuclei in the SV; total number of cells per SV was extracted

**Fig. 3:**
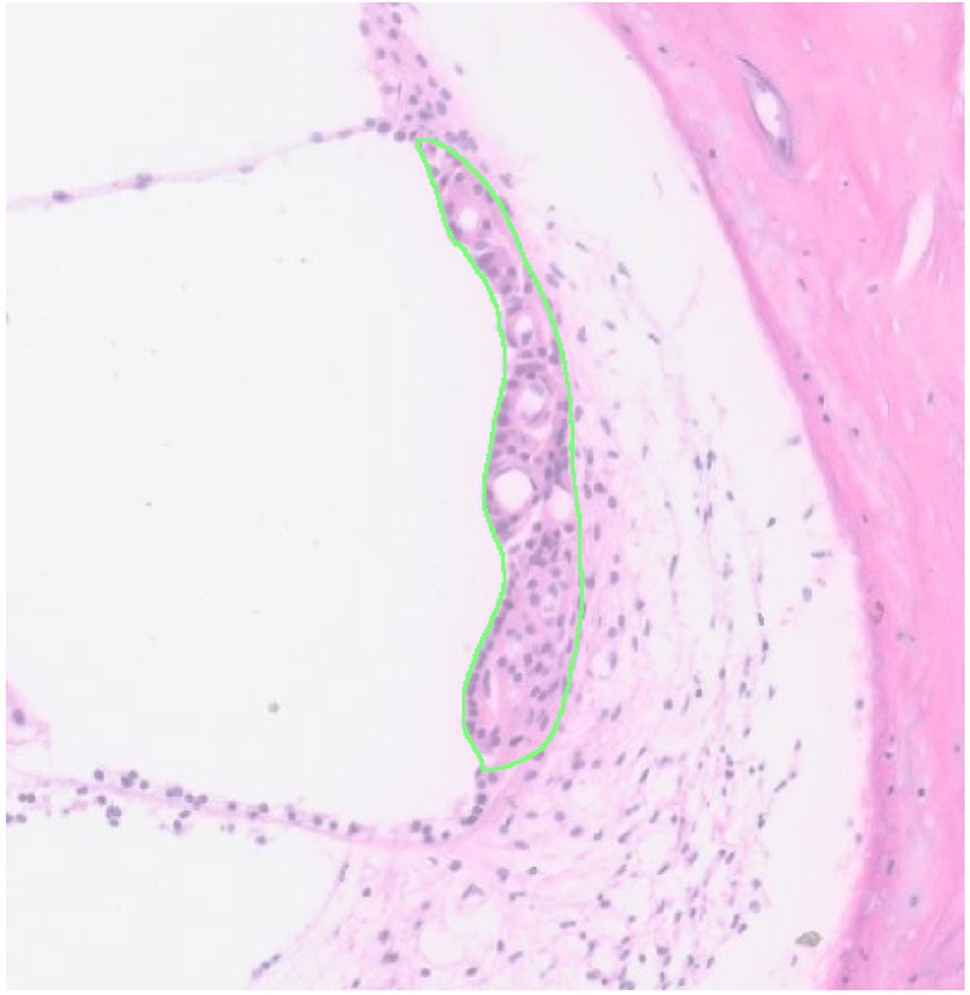
Width calculation from SV; w1, the minimum Feret diameter; w2, intersection of SV area and the midline of the bounding box of SV; w3, area of SV (*a*)/height of bounding box (*h*)

As part of SVPath, SVAT was developed to extract features of the SV; an overview of these features is shown in Fig. 2. As noted in other studies, the morphology of the SV is analyzed primarily by measuring its width and cross-sectional area [2, 3, 7, 10]. SVAT uses the binary mask of the SV generated from the previous step to calculate the pixel area that was later converted into micrometers [2]. Conversion from pixels to micrometers was done by correlating measurements of features within QuPath to pixels in image. The width of the SV is measured in three different ways, as shown in Fig. 3: minimum Feret diameter (w1), width along the union of the midline of the bounding box and the SV mask(w2), and average width (w3). The average width is defined by the formula below.

$$Average\; width=\frac{Area\; of\; SV}{Height\; of\; SV}$$

The height of the SV is computed from the bounding box around the SV mask. All units are reported in pixels, later converted into micrometers.

The binary mask of the capillary bed is processed to extract the number of capillaries found in the SV, the total area of the capillary lumen per SV, and the average area per capillary measured. The final metric reported by software is the number of nuclei found in the SV. The number of nuclei is calculated by first taking the original 512 × 512 color SV patch and performing K-means image clustering to segment the nuclei within the SV. These nuclei are then analyzed to determine the average area of a single nuclei in a patch. Then, the total nuclei area is divided by the average area of a single nuclei in the patch to estimate the total number of nuclei in each patch. This method for calculating the number of nuclei has been validated in other studies [18]. While there are some multi-nucleated, irregular cells within the SV, counting the number of nuclei can estimate the number of cells [19].

### Training and Evaluation of [YOLOv8]^2^ and nnUnet

To train the YOLOv8 neural network for cochlea detection, a smaller random data set of 110 compressed square WSI (2048 × 2048 px) from macaque M1 was manually labeled with a bounding box around the cochlea using Roboflow, an easy-to-use machine learning platform [20]. Labeling was performed by three separate raters. Once these slides were labeled, the data was randomly partitioned into a training and validation set with an 80/20 split. Using the training subset, a PyTorch implementation of YOLOv8 neural network was trained on a virtual graphics processing unit (GPU) and evaluated on the validation set. The training was run for 30 epochs. Three performance metrics were used to assess YOLOv8:

1. Precision: Positive predictive value of the bounding box generated by neural network compared to the labeled ground truth.
2. Recall: Sensitivity of the bounding box generated by neural network compared to the labeled ground truth.
3. mAP: An average of the precision and recall per class labeled across all the images in the validation set.

Performance metrics were captured specifically for the internal validation dataset. A similar process was done for the YOLOv8 neural network for SV detection, except 78 compressed square images of the cochlea region (2048 × 2048 px) were used for the dataset, and the SV region was labeled. The performance metrics used to evaluate the Yolov8 neural network are consistent with other literature [23].

Once the SV region was extracted into a 512 × 512px patch, nnUnet was used to segment the SV and its associated capillaries. The nnUnet was trained and validated on 220 patches extracted from the M1 ear using the [YOLOv8]^2^ method described above. The SV and its associated capillary bed in each of these patches were manually labeled using RoboFlow. During the training process, the dataset was randomly partitioned into five folds; each fold randomly divided the 220 patches into training and validation subsets with an 80/20 split. The nnUnet was trained on each fold separately for 50 epochs. Optimizable parameters that maximize the performance of nnUnet were determined based on the individual performance of each of these folds. To evaluate nnUnet performance, two similar metrics were used:

1. Dice score (i.e., dice similarity coefficient, DSC): The overlap between the predicted segmentation and the ground truth by computing the intersection of the two masks and dividing it by the average of their areas. A higher dice score indicates better alignment between prediction and truth, with one being a perfect match.
2. Intersection over union (IoU): The ratio of the intersection to the union of the predicted and ground truth masks. An IoU score of 1 represents a perfect match.

Dice and IoU are commonly used metrics used to evaluate segmentation neural networks [24]. Both capture similar metrics; however, IoU is more significantly affected by incorrect segmentation-compared to dice scores, resulting in lower scores. In previous literature, dice values greater than 0.7 are considered acceptable segmentation performance [11]. Performance metrics were captured for the entire dataset.

### External Validation on Macaque Ear M2

In addition to validating this method on the M1 ear, a second normal macaque ear, M2, was used to increase the generalizability of this model. The dataset of M2 ear consisted of 7–8 WSI from the mid-modiolar region within the cochlea with approximately 20 SV. Each SV with its associated capillaries was manually labeled and compared with labels generated using the SVPath tool outlined above. Additionally, the generated labels were analyzed using the SV tool to demonstrate how this tool could be used to compare different ears.

## Results

**Fig. 4:**
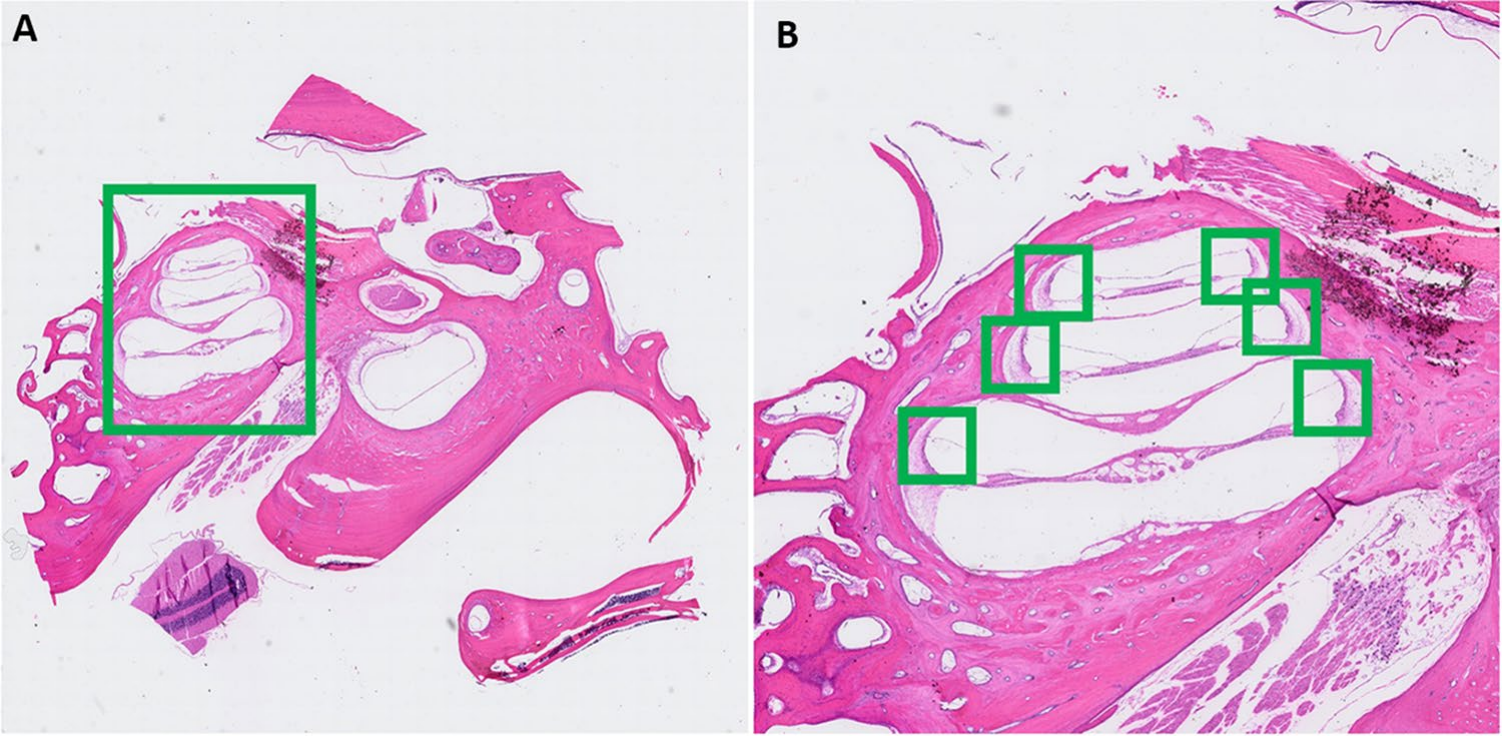
Example of object detection task performed by YOLOv8; A detection of cochlea from whole slide image; B detection of SV from the cochlear region

**Fig. 5:**
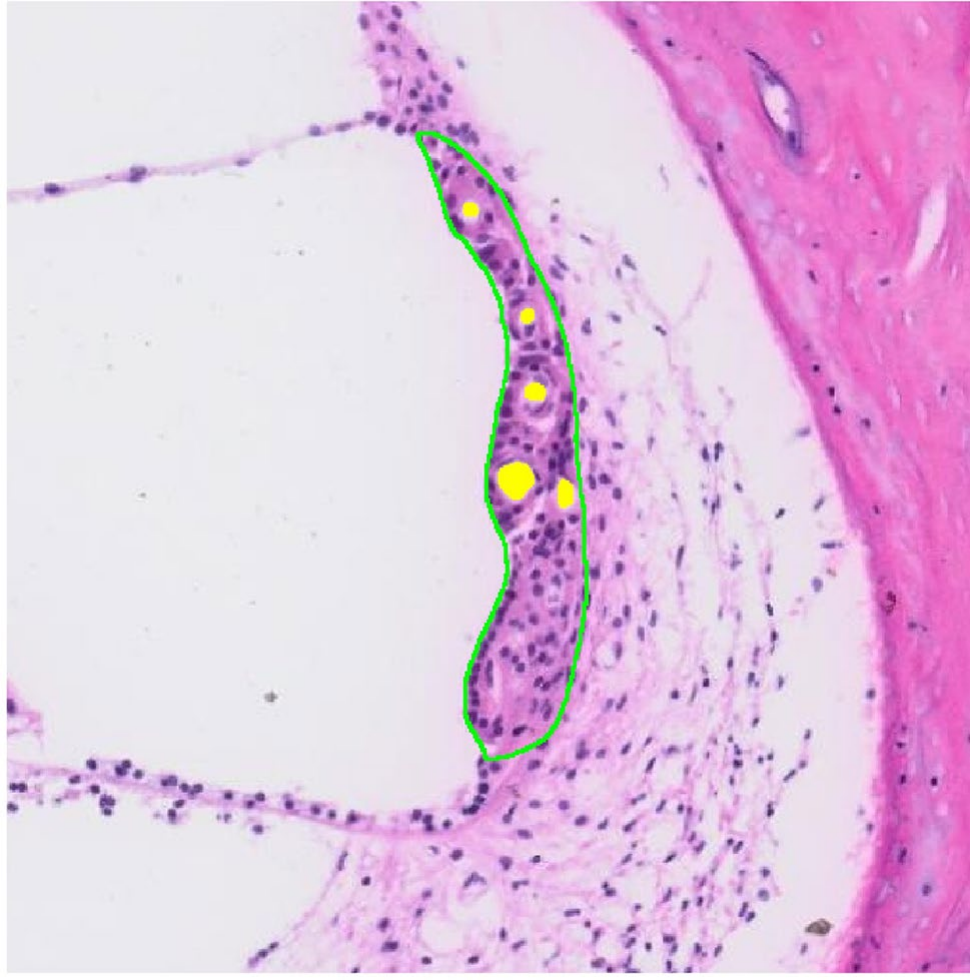
Example of results from nnUnet segmentation; green region corresponds to SV and yellow represents associated capillaries

Qualitative assessment of [YOLOv8]^2^ and nnUnet training and validation is shown in Figs. 4 and 5, respectively. Applying both steps took an average of ~ 40 s per WSI. As mentioned above, the performance of [YOLOv8]^2^, the object detection portion of the model, is measured by mAP, precision, and recall. These metrics were captured for detecting the cochlea and the SV for both macaque ear M1, the internal dataset, and M2, the external dataset, and presented in Table 1. On average, the mAP for cochlea and SV detection for the internal dataset was 97.5%, which fell slightly to 96.4% for the external dataset. The average precision and recall for both structures in the internal dataset were 94.1% and 96.8%, respectively, slightly higher than the external dataset (average precision/recall: 92.7%/95.6%). Overall, performance across all metrics was greater than 90% for both structures across the internal and external datasets.

**Table 1:** Performance of YOLOv8 object detection for the cochlea and SV

	**Internal validation dataset**		**External validation dataset**	
	Detect cochlea (*n* = 22)	Detect SV (*n* = 16)	Detect cochlea (*n* = 10)	Detect SV (*n* = 10)
mAP	97.1%	98%	95.8%	96.8%
Precision	91.7%	96.5%	93.1%	92.2%
Recall	97.4%	96.3%	93.8%	97.4%

Metrics to assess the performance of the nnUnet included average dice score and IoU. A score of 1 indicates a perfect overlap between the ground truth mask and the mask generated by nnUnet. Average SV segmentation was more accurate than segmentation of the CB in both internal (SV: 0.95, CB: 0.88) and external (SV: 0.84, CB: 0.75) validation datasets (Table 2). All the SV and most CB segmentations met the acceptable criteria dice score > 0.7 for both the internal and external datasets.

**Table 2:** Performance of nnUnet network for segmentation of SV and its associated capillary bed (CB) for internal and external validation

	**Internal validation dataset (n=220)**		**External validation dataset (n = 20)**	
	Segment SV	Segment CB	Segment SV	Segment CB
Mean dice [min–max]	0.95 [0.98–1.00]	0.88 [0.58–1.00]	0.84 [0.82–0.95]	0.75 [0.51–0.90]
IoU	0.91	0.79	0.75	0.61
“Acceptable” criteria (dice > 0.70)	100%	92%	100%	80%

*IoU *intersection over union

**Fig. 6:**
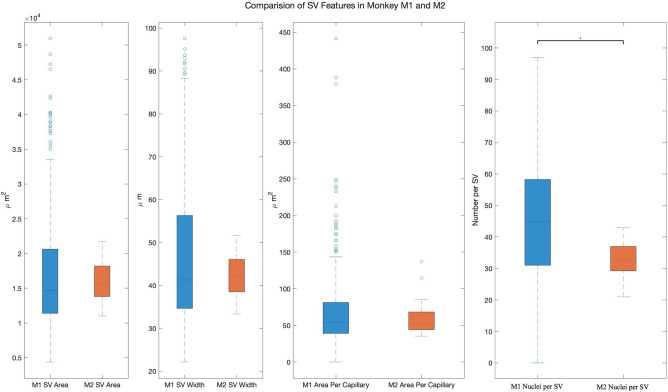
Example of comparison of SV including SV area, SV width, area per capillary, and number of nuclei per SV in monkeys M1 and M2. There was a statistical difference between M2 and M1 number of nuclei in SV

Figure 6 demonstrates how SVAT could be applied to compare two ears. The M1 and M2 macaque ears represent normal monkey ears stained and sectioned using a similar protocol. There was no statistical difference between M1 and M2 SV area, SV width, or area per capillary (*p* = 0.48, *p* = 0.34, and *p* = 0.42, respectively). However, a statistical difference was noted for nuclei per SV between the M1 and M2 (*p* < 0.001).

## Discussion

The stria vascularis is integral to normal cochlear physiology and may play a role in several otologic pathologies. However, further understanding of this structure has been in part limited by the shortcomings of manual segmentation and analysis of histopathological slides. The SVPath pipeline that we have developed leverages novel methods in deep learning to facilitate accurate and high-throughput extraction and analysis of the SV from temporal bone WSI.

The validation results for the [YOLOv8]^2^ method demonstrate that using two separate YOLOv8 neural networks in series can successfully extract the location of small structures, such as the SV, from within a large WSI. A high degree of accuracy for detecting the cochlea from within the WSI and the SV from within the cochlea region was expected since using the YOLO neural network framework to detect objects has previously been validated [15]. Similarly, the validation results for nnUnet indicate that SVPath can successfully segment the SV and its capillary bed from patches extracted by [YOLOv8]^2 ^[16]. The difference in performance between SV and capillary bed relates to a few factors. Generally, neural networks for image segmentation perform better when more pixels represent a label relative to the background. Given that the SV is represented by significantly more pixels than the CB within any given patch, a decrease in performance is expected. Additionally, the capillary bed’s shape, size, and other features are more variable than the SV. Generating more training data to capture this variability may lead to better performance. While SV segmentation outperformed capillary bed segmentation, most segmented capillary beds still met the acceptable criteria (dice score > 0.7). Both the YOLOv82 and the nnUnet, when applied to an external dataset consisting of a separate macaque ear specimen not used for training and internal validation, achieved similar performance across both tasks, indicating that this method is generalizable.

Regarding the SV analysis, given that both the external and internal datasets were collected from normal ears, a lack of a statistically significant difference between the datasets for the metrics analyzed was expected. However, the number of nuclei was found to be different between the two ears. This may be due to the difference in size between the two datasets; only 20 SV were analyzed for the M2 ear compared to 220 SV for the M1 ear. The purpose of including SVAT results was to demonstrate how it can extract clinically relevant features from the SV. Future work analyzing more sections across various normal macaque ears is required to generalize these findings.

Successful validation of SVPath to analyze SV has clinical implications. As mentioned above, SV plays a role in many ear diseases; however, the pathophysiology remains poorly understood. For instance, contrary to convention, Wu et al. suggested that SV atrophy was a secondary effect of presbycusis rather than its cause [21]. Since then, other groups, including Lang et al., have suggested that SV may be involved early in the pathogenesis of presbycusis [22]. In both studies, the groups manually sampled the SV at a few sites per slide for analysis and used a single metric (SV cross-sectional area vs. SV thickness). By automating the analysis, SVPath facilitates large-scale, complete SV analysis that may lead to insights about the role of SV in presbycusis and other diseases. SVPath allows researchers to capture metrics to quantify SV atrophy in multiple ways and focus on the vasculature within the SV. Further, SVPath, by creating a standard method to analyze SV, avoids the pitfalls of inter-rater variability in manual segmentation.

Other groups have segmented and analyzed WSI using neural networks. Khened et al. proposed a generalized framework that used an ensemble of neural networks based on a U-net with various backbones to improve the model’s overall accuracy [12]. Guo et al. created quick yet accurate deep-learning methods to analyze breast images, where they used a patch-based method to extract cancerous regions and then evaluated these regions at different resolutions to gather course and fine features [13]. Both methods offer accurate WSI segmentation, but the SVPath pipeline offers two significant advantages. The proposed pipeline captures features at the level of the WSI, like the cochlea, and features at the level of the cochlea, like SV. This pipeline could easily extend to extract features ranging from relatively large structures like semicircular canals to finer structures like the tectorial membrane. Additionally, this pipeline preserves global location information of the SV relative to the original WSI, allowing researchers to overlay structures on the WSI. Using existing image registration methods, stacked, aligned WSI can generate 3D models of histologic structures from temporal bone slides. Such information could further lead to understanding of how complex spatial relationships contribute to various pathologies. There may be other proprietary deep learning pathology tools that function like SVPath; however, these tools can be financially prohibitive. SVPath is built on other open-source tools, thereby enabling increased access such technology.

There are some limitations of the SVPath tool. While SVPath was validated on an external dataset, this dataset was stained and sectioned similarly to the training data. Uniformity of staining for both datasets was ensured by manually inspecting each image processed. Adding and training with differently stained datasets or datasets sectioned in other planes in the future could further improve the generalizability of this tool. Additionally, this tool was limited to training and testing on healthy macaque ears; future work should focus on applying this technique to diseased ears and human temporal bone specimen WSI to improve the clinical utility. From a technical standpoint, training multiple neural networks was computationally expensive, requiring a dedicated GPU. Additionally, preserving images at multiple levels requires significant memory and storage. Despite these disadvantages, the proposed SVPath tool has the potential to improve current analysis techniques for the SV dramatically.

## Conclusion

SVPath provides a deep learning pipeline for automatic analysis of SV. This pipeline can be extended to other structures within temporal bone WSI and facilitate 3D analysis of such structures. As the digital temporal bone WSI database grows, integrating such tools will enable researchers to conduct rapid, large-scale investigations of structures within temporal bone WSI. Such studies could translate to new insights into the mechanism of various otologic pathologies and guide future interventions.

## Data Availability

Some sample macaque ear data to test the code has been released to validate the code developed here (https://drive.google.com/drive/folders/1bAalpF6-O6Vj1dI_oV2VEaTYHjhd_BuM?usp=share_link). Additional data can be requested by contacting Dr. Amanda Lauer (alauer2@jhmi.edu).

## Code Availability

The code used to analyze the SV has been released on GitHub (https://github.com/2014ajain/StriaVascularis).
